# Effect of Adhesive Pretreatments on Marginal Sealing of Aged Nano-ionomer Restorations

**DOI:** 10.15171/joddd.2015.028

**Published:** 2015-09-16

**Authors:** Fereshteh Shafiei, Sahar Akbarian, Mohammad Karim Etminan

**Affiliations:** ^1^Professor, Prevention of Oral and Dental Disease Research Center, Department of Operative Dentistry, School of Dentistry, Shiraz University of Medical Sciences, Shiraz, Iran; ^2^Assistant Professor, Department of Operative Dentistry, Faculty of Dentistry, Shiraz University of Medical Sciences, Shiraz, Iran; ^3^Undergruduate Student, Faculty of Dentistry, Shiraz University of Medical Sciences, Shiraz, Iran

**Keywords:** Acid etching, self-etch adhesive, etch-and-rinse adhesive, microleakage, nano-ionomer

## Abstract

***Background and aims. ***Nano-ionomer (NI) interacts with tooth structures superficially, and there is a concern about the enamel bonding ability of mild self-etch Ketac primer. This study compared the effect of different adhesive procedures (self-etching and etch-and-rinse approach) on long-term marginal microleakage of nano-filled resin-modified glass-ionomer (NI) cervical restorations.

***Materials and methods.*** Class V cavities were prepared on 72 maxillary premolars. The teeth were divided into six groups: G1: No treatment (NC); G2: Ketac primer (K primer); G3: Etchant + Ketac primer (E+K primer); G4: Self-etch adhesive (Bond Force); G5: Etchant + Bond Force (E+Bond Force); G6: Etchant + Adper Single Bond (Etch and rinse adhesive). All the cavities were restored with Ketac N100. The samples were stored in water for 6 months and thermocycled for 2000 cycles. Marginal sealing was assessed using dye penetration technique. Data were analyzed with non-parametric tests (α=0.05).

***Results.*** All the adhesive pretreatments resulted in a lower marginal leakage than that of NC (P≤0.01), except for E+Bond Force at the dentin margin. There was no significant difference between K primer and Bond Force. Microleakage differed significantly between K primer pretreatment and E+K primer (P=0.003), E+Bond Force (P=0.002) and etch-and-rinse adhesive (P=0.001) at the enamel margin, but it did not differ at the dentin margin. E+ Bond Force group showed insignificantly lower leakage at the enamel margin and significantly higher leakage at the dentin margin (P=0.02).

***Conclusion.*** Etch-and-rinse adhesive and selective enamel etching along with self-etch adhesive/Ketac primer might improve marginal sealing of aged nano-ionomer restoration.

## Introduction

Resin-modified glass-ionomers (RMGIs) are a combination of glass-ionomer cement and resin components.^1,2^ These materials have numerous advantages, including dual-curing setting reaction, bonding ability to tooth structures through a two-fold bonding mechanism, increased mechanical and esthetic properties, improved handling and working characteristics and reduced moisture or dehydration sensitivity.^1-5^

A novel, highly packed, nano-filled RMGI, Nano-ionomer (NI, Ketac N100) has been introduced to the dental market to restore small cavities. Incorporation of approximately 69% of filler by weight or 56% by volume, of which two-thirds are nano-fillers, has improved mechanical properties, wear resistance, color and polishability characteristics and resistance to biomechanical degradation.^6-8^ One of the disadvantages of RMGI is that they are degradable and may deteriorate faster than the bond to dentin. However, NI seems to be more resistant to water degradation.^9^

When comparing abrasion resistance, NI behaved as an intermediate material between RMGI and nanocomposite because it had an intermediate composition.^10^ In addition, this high-filled RMGI exhibited lower polymerization shrinkage and lower coefficient of thermal expansion and fluoride release compared to RMGI.^11,12^ The two-paste mixing system of NI might facilitate its handling characteristics.^7,8^These advantageous properties might result in the widespread use of NI instead of RMGI in clinical situations.

However, NI, in contrast to RMGI, has no self-adhesive capacity and the use of Ketac Nano primer before NI is an essential step to its bond to tooth structures. This self-etch primer (pH=3) contains the acrylic/itaconic acid copolymer dissolved in HEMA and water; its low acidity may not allow the primer to totally dissolve/remove the smear layer.^11^ NI interacts very superficially with the dentin and enamel without demineralization or hybrid layer formation. This interaction provided micromechanical interlocking into the substrate roughness that is most likely supported by chemical bonding of polyalkenoic acid copolymer with surface hydroxyapatite.^11^ However, in addition to chemical bonding, RMGI could establish micromechanical bonding of the monomeric component into partially demineralized smear layer-free tooth surfaces.^4,12^

According to TEM observations, NI revealed a tight interface with the tooth structure. Nevertheless, despite its effective bonding, it bonded less effectively than RMGI after 24 hours.^11^

The chemical bonding might have played an important role in the stability of marginal integrity and excellent performance of RMGI in a clinical trial conducted by Peumans et al.^13^ The chemical bonding is achieved through acid-base reaction at the adhesive interface of RMGI.^8,14^

On the other hand, the higher filler and resin content of NI compared to RMGI was reported to possibly result in a reduced acid-base reaction.^15^ The reduced enamel marginal adaptation of NI cervical restorations was reported after one year of clinical service.^15^

A number of studies have reported that the use of different self-etch adhesives could improve dentin bonding/sealing of RMGI.^16-18^ On the other hand, some authors indicated that acid etching of the enamel could increase the bond strength of RMGI.^19^

A recent study has indicated that using self-conditioner and especially an etch-and-rinse adhesive instead of cavity conditioner for RMGI (Fuji II LC) and Ketac primer for NI (Ketac N100) could improve the bond strength to dentin.^20^

Therefore, this study was designed to evaluate the effects of different adhesive cavity pretreatments (self-etch adhesive and Ketac primer with or without an additional etching and an etch-and-rinse adhesive) on the long-term sealing ability of NI cervical restoration. The null hypothesis was that prior different adhesive procedures have no effect on the marginal sealing of Class V NI restorations after water aging.

## Materials and Methods

The research protocol was approved by the local ethics committee. Seventy-two sound human maxillary premolars recently extracted for orthodontic treatments were selected, cleaned and disinfected in 0.5% chloramine solution for two weeks. The teeth were then stored in distilled water at 4°C, until use in the experiment. Standard Class V cavities (3 mm in width, 3 mm in height and 1.5 mm in depth) were prepared at the CEJ on the buccal surface with the gingival margin in dentin and the occlusal margin in enamel. The teeth were randomly divided into six groups of 12 each, according to different adhesive cavity pretreatments as follows:

Group 1 (NC): Negative control with no treatment

Group 2 (K Primer): Pretreatment with Ketac primer

Group 3 (E+K primer): Pretreatment with phosphoric acid etching + Ketac primer

Group 4 (Force Bond): Pretreatment with Force Bond self-etch adhesive

Group 5 (E+Force Bond): Pretreatment with phosphoric acid etching + Force Bond

Group 6 (Etch-and-rinse adhesive): Pretreatment with acid etching + Adper Single Bond.

The materials used and their application procedures are presented in Table 1. Following surface pretreatment, all the cavities were restored using a nano-ionomer (NI), Ketac N100 (3M ESPE, USA), in two increments, each light-cured for 30 seconds according to the manufacturer’s instructions. The restorations were finished and polished with Sof-Lex discs (3M ESPE). The teeth were stored in distilled water at 37°C for six months (the water was changed every week) and then subjected to 2000 thermal cycles at 5/55°C in water baths with a 30-second dwell time and a 15-second transfer time. The root apices were sealed with utility wax, and all the surfaces except for the restorations and 1 mm from the margins were coated with two layers of nail varnish. The teeth were immersed in a 0.5% methylene blue dye solution for 24 hours. They were then rinsed thoroughly in tap water, blot-dried and sectioned through the center of the restorations from the facial to the lingual surface with a water-cooled diamond wheel saw (Leitz 1600, Wetzlar, Germany). The sections were assessed in a blind manner for dye penetration by two evaluators under a stereomicroscope (Carl Ziess Inc, Oberkochen, Germany) at ×20 magnification. Dye penetration at the restoration-tooth interface was scored for the enamel and dentin margins according to standardized criteria:

**Table 1:** Materials used in this study

**Material/Manufacture**	**Composition/Batch**	**Application Procedure**
Bond Force/Tokuyama Dental, Tokyo, Japan	Phosphoric acid monomer, Bis-GMA, HEMA, TEGDEMA, Comphorquinone, alcohol, water/106MM pH=2.3	Apply adhesive with gently rubbing for 20 seconds, dry gently for 5seconds then strongly for 5 seconds. Light cure for 10 second
Ultra-Etch/Ultradent, South Jordan, Utah, USA	35% Phosphoric Acid /B5Y7M	Apply acid gel for 15 seconds, water rinse for 20 seconds, dry gently
Ketac Primer/3M ESPE, St Paul, MN, USA	HEMA, water, Vitrebond copolymer, photoinitiator/N251185 pH=3	Apply for 15 seconds, air dry for 10 seconds, light cure for 10 seconds
Ketac N 100/3M ESPE, St Paul, MN, USA	HEMA, vitrebond copolymer, water, TEGDMA, PEGDMA, BisGMA, fluoroaluminosilicate glass, silane-treated zirconia/ silica, photoinitiators/N271282Bis-GMA,HEMA,dimethacrylates, polyalkenoic acid copolymer, initiator, water, ethanol/N266218	Mix two pastes, insert into cavity, light cure
Adper Single Bond/3M ESPE, St Paul, MN, USA	Bis-GMA, HEMA, dimethacrylates, polyalkenoic acid copolymer, initiator, water, ethanol/N266218	Apply two consecutives coats of adhesive, air dry gently for 2-5 seconds, light cure for 10 seconds

Score 0: No leakage visible at the tooth‒restoration interface

Score 1: Penetration of dye along the cavity wall, but less than one-half of the length

Score 2: Penetration of dye along the cavity wall, but short of the axial wall

Score 3: Penetration of dye to and along the axial wall

The worst score from the two sections of each specimen was recorded. The microleakage data were analyzed using Kruskal-Wallis and Mann-Whitney statistical tests at a significance level of 5%.

## Results

Dye penetration scores for the enamel and dentin margins in the six groups are presented in Tables 2 & 3. According to the results of Kruskal-Wallis test, there were the same significant differences among the six groups at the enamel and dentin margins (P<0.001). Pair-wise multiple comparisons of the six groups revealed that all the groups had significantly lower microleakage than that of NC at both margins (P≤0.01), except for group 5 at the dentin margin (P=0.14).

**Table 2:** Dye penetration score frequencies at enamel margins for the six study groups

**Groups**	**Microleakage Scores**				**Median**
	**0**	**1**	**2**	**3**	
**1. NC**	0	0	3	9	3
**2. K Primer**	1	3	4	4	2
**3. E+K Primer**	9	0	3	0	0
**4. Force Bond**	4	1	4	3	2
**5. E+Force Bond**	8	2	2	0	0
**6.Etch-and-rinse Adhesive**	9	1	2	0	0

**Table 3:** Dye penetration score frequencies at dentin margins for the six study groups

**Groups**	**Microleakage Scores**				**Median**
	**0**	**1**	**2**	**3**	
**1. NC**	0	1	4	7	3
**2. K Primer**	5	3	3	1	1
**3. E+K Primer**	7	2	1	2	0
**4. Force Bond**	8	3	0	1	0
**5. E+Force Bond**	3	1	4	4	2
**6. Etch-and-rinse Adhesive**	9	2	1	0	0

No significant differences were found between groups 2 and 4, indicating approximately similar microleakage for K primer and Bond Force at both margins. At the enamel margin, there were significant differences between group 2 and groups 3, 5 and 6 (P=0.003, P=0.002 and P=0.001, respectively), revealing a positive effect of acid etching on reducing microleakage; however, there were no significant differences at dentin margins. Comparison of groups 4 and 5 showed an insignificant lower leakage for group 5 than group 4 at the enamel margin, but a significantly higher leakage for group 5 (E+Bond Force) than group 4 (Bond Force) at the dentin margin (P=0.02). Group 6 (etch-and rinse adhesive) exhibited the lowest microleakage at both margins that was not different from group 4 at the dentin margin, while it was significantly different from group 4 at the enamel margin (P=0.03).

## Discussion

Maintaining the enamel and dentin margins of cervical restorations sealed against microleakage remains a major factor in clinical longevity.^21^ In the current study, Ketac N100 restorations were subjected to thermocycling and water storage to challenge marginal integrity over time. The thermal fatigue stress and hydrolytic effect of water on the adhesive interface simulate conditions in the oral cavity, thereby predicting the behavior of the aged adhesive interface.^22^According to the results of this study, all the adhesive pretreatments significantly improved marginal sealing of the aged NI restorations compared to that of negative controls (with no treatment), except for E+Bond Force in which this improvement was not significant at the dentin margin. Ketac primer might act as a mild self-etch adhesive due to acidic monomers and photoinitiator content; it may create a resin covering on the primed surfaces after light-curing.^15^

Consistent with concerns about the long-term marginal sealing effectiveness of self-etch adhesives to the enamel,^23^ our results indicated relatively high microleakage at the enamel margin of aged NI restorations. Microleakage was significantly higher than at the dentin margin. Because of its low acidity, Ketac primer may not be able to interact sufficiently with the highly mineralized enamel structure. Recently, unstable bonding and increased microleakage with time were reported at the enamel margin of NI restorations.^11^ In addition, a one-year clinical trial of NI restorations disclosed enamel marginal deficiencies. These authors suggested that this decreased marginal adaptation was similar to the results obtained with self-etch adhesives in clinical evaluations.^15^

The self-etch adhesive, Bond Force used in this study, did not perform significantly better than Ketac primer in terms of marginal sealing. This self-etch adhesive (pH=2.3) contains a phosphoric acid monomer. It seems that despite the lower pH of Bond Force than Ketac primer, this adhesive was not capable of significantly lowering microleakage at both margins. In a recent study, the use of four self-etch adhesives prior to NI was reported to provide a higher or similar bond strength compared to that of Ketac primer, depending on their chemical compositions.^24^ Imbery et al^20^ reported a higher bond strength of self-conditioner/NI compared to Ketac primer/NI to dentin. These authors suggested that the lower pH of self-conditioner (1.8) might have more completely removed the smear layer compared to Ketac primer.

In a new study conducted by Shafiei and Akbarian,^25^ using Silorane Adhesive System (pH=2.7) and Clearfil SE Bond (pH=2) instead of Ketac primer prior to NI could significantly improve early cervical sealing in open modified sandwich restorations.

On the other hand, resin content of NI can create direct covalent bonds with the resin layer formed on the surface of self-etch adhesive, similar to that of RMGI.^17^ The water content in both NI and self-etch adhesive compositions might lead to compatibility between them.

The results of the present study indicated that an additional etching prior to Ketac primer improved sealing at the enamel margin but had no effect on dentinal marginal sealing in aged restorations. Consistent with our results, Glasspoole et al^19^ reported an early increase in bond strength of an RMGI to the enamel after using phosphoric acid.

Although acid etching prior to RMGI use was reported to provide better resin penetration into the underlying dentin and consequently better hybridization, this micromechanical interlocking might not benefit from additional chemical bonding.^9^ It is well documented that this bonding plays a decisive role in marginal sealing and bonding durability.^9,26^ The thicker hybrid layer on acid-etched dentin observed in a TEM analysis by Coutinho et al^27^ in combination with a low-molecular-weight self-etching polymer resulted in greater early bond strength with an RMGI. Nevertheless, the lack of intimate interaction with hydroxyapatite-coated collagen led to instability of the interface in the long term.^9^ The same point may account for the higher microleakage obtained at the dentin margin of cavities in which acid etching was used before Bond Force/NI. However, this procedure led to an insignificant decrease in microleakage at the enamel margin (P=0.06). So far, no study has been performed on the effect of prior acid etching on bonding or sealing ability of Ketac primer/NI or self-etch adhesive/NI.

The beneficial effect of additional acid etching on the bonding efficacy of some self-etch adhesives/composite resin to the enamel^28-30^ and even to the dentin^31,32^ was previously reported. However, some authors have proposed that acid etching of the dentin may reduce the adhesive potential of self-etch adhesives.^28-30^ It, therefore, appears that the bonding ability of self-etch adhesives to the etched dentin may be adhesive-specific.^31,32^ This assumption was relevant in the present study. The acid etching of the dentin prior to Bond Force increased the marginal leakage, whereas it displayed no effect on marginal leakage for Ketac primer.

In fact, enamel etching capability of self-etch adhesives is controversial. So, some authors have advised selective enamel etching to improve enamel bonding.^28-30^ Our results supported this recommendation, in particular for self-etch Ketac primer whose insufficient acidity is not capable of achieving reliable enamel bonding. The positive effect of selective enamel etching on the adhesive properties of Bond Force was indicated by a two-year clinical study.^33^

According to our findings, pretreatment with an etch-and-rinse adhesive compared to Ketac primer significantly reduced microleakage at the enamel margin but this reduction was not significant at the dentin margin. Etch-and-rinse adhesive/NI yielded the best performance in terms of preventing marginal leakage after aging.

This finding is in line with a study by Bayrak et al,^34^ demonstrating positive effect of etch-and-rinse adhesive on early marginal sealing of an RMGI (Vitremer). In a recent study, the highest bond strength of two RMGIs and NI to the dentin was attained by using an etch-and-rinse adhesive in short term.^20^ These results possibly supported the important role of the hybrid layer and resin tags in providing bonding durability, especially at the enamel margin. In addition, establishment of chemical bonding via using self-etch adhesive with partly demineralized dentin may be beneficial in terms of marginal sealing stability.^9^ However, etch-and-rinse adhesives were reported to have no effect on bonding of two RMGIs; the bonding may be more material-dependent than surface treatment-dependent.^35^

It was reported that dentin pretreatment using a HEMA-rich primer may compromise the formation of the absorption layer, and the acid-base reaction continuation in RMGI at the adhesive interface.^36^

Applying an etch-and-rinse adhesive prior to NI could lead to the formation of a hybrid layer into demineralized dentin and an adhesive layer on the dentin surface. Consequently, any chemical bonding of NI was not expected. However, a good union could be established between polyacrylic acid copolymer and resin content of both Adper Single Bond and NI. In this situation, bonding mechanism of NI to dentin is more like a composite resin than a glass-ionomer and the sealing ability of NI depends on the hybrid layer formation.

Clinically, one advantage of using an etch-and-rinse or self-etch adhesive as surface pretreatment might be facilitation of the bonding procedures with simultaneous adhesive application for both NI and composite resin in a sandwich restoration. However, a decrease in fluoride release from NI following formation of an adhesive layer on the treated surface might be considered as a disadvantage. Nevertheless, clinicians would take advantage of the released fluoride from NI in the proximal surfaces of the adjacent teeth in an open sandwich restoration.

Further studies of long-term bond strength and scanning electron microscopy analysis are required to confirm the results of the present study. Furthermore, clinical trials should be conducted on the long-term efficacy of different adhesive pretreatments for NI restorations.

## Conclusion

Under the limitations of this in vitro study, using etch-and-rinse adhesive and acid etching along with Ketac primer might be suggested in terms of marginal sealing of aged NI cervical restorations. However, when using a self-etch adhesive, selective enamel etching is recommended.

## Acknowledgement

The authors wish to thank the Vice-Chacellery of Shiraz University of Medical Sciences, Shiraz, Iran, for supporting this research (grant no. 92-5666); Dr Mehrdad Vossoughi, Assistant Professor, Center for Research Improvement, School of Dentistry, Shiraz University of Medical Sciences, for statistical analysis, and Dr Nasrin Shokrpour for help with the English language. This article is based on the thesis by M K. Etminan.

## References

[1] Mitra SB (1991). Adhesion to dentin and physical properties of a light-cured glass-ionomer liner/base. J Dent Res.

[2] Sidhu
 SK
, Watson
 TF
 (1995). Resin-modified glass ionomer materials: A status report for the American Journal of Dentistry. Am J Dent.

[3] Xie D, Brantley WA, Culbertson
 B
, Wang
 G
 (2000). Mechanical properties and microstructures of glass-ionomer cements. Dent Mater.

[4] Abdalla AI (2000). Morphological interface between hybrid ionomers and dentin with and without smear-layer removal. J Oral Rehabil.

[5] Bourke AM, Walls
 AW
, Mc Cabe
 JF
 (1992). Light-activated glass polyalkenoate (ionomer) cements: the setting reaction. J Dent.

[6] Mitra SB, Wu D, Holmes BN (2003). An application of nanotechnology in advanced dental materials. J Am Dent Assoc.

[7] Killian CM, Croll TP (2010). Nano-ionomer tooth repair in pediatric dentistry. Pediatr Dent.

[8] Coutinho E, Cardoso MV, De Munck J, Neves AA, Van Landuyt KL, Poitevin A (2009). Bonding effectiveness and interfacial characterization of a nano-filled resin-modified glass-ionomer. Dent Mater.

[9] De Munck J, Van Meerbeek B, Yoshida Y, Inoue S, Suzuki K, Lambrechts P (2004). Four-year water degradation of a resin-modified glass-ionomer adhesive bonded to dentin. Eur J Oral Sci.

[10] Carvalho FG, Sampaio CS, Fucio SBP, Carlo HL, Correr-sorbino L, Puppin-rotani RM (2012). Effect of chemical and mechanical degradation on surface roughness of three glass ionomers and a nanofilled resin composite. Oper Dent.

[11] Abd El Halim S, Zaki D (2011). Comparative evaluation of microleakage among three different glass ionomer types. Oper Dent.

[12] Coutinho E, Yoshida Y, Inoue S, Fukuda R, Snauwaert J, Nakayama Y (2007). Gel phase formation at resin-modified glass-ionomer/tooth interfaces. J Dent Res.

[13] Peumans M, Kanumilli P, De Munck J, Van Landuyt K, Lambrechts P, Van Meerbeek B (2005). Clinical effectiveness of contemporary adhesives: a systematic review of current clinical trials. Dent Mater.

[14] Cardoso MV, Delmé KI, Mine A, Neves Ade A, Coutinho E, De Moor RJ (2010). Towards a better understanding of the adhesion mechanism of resin-modified glass-ionomers by bonding to differently prepared dentin. J Dent.

[15] Perdigão J, Dutra-Corrêa M, Saraceni SH, Ciaramicoli MT, Kiyan VH (2012). Randomized clinical trial of two resin-modified glass ionomer materials: 1-year results. Oper Dent.

[16] Pereira PNR, Yamada T, Inokoshi S, Burrow MF, Sano H, Tagami J (1998). Adhesion of resin-modified glass ionomer cements using resin bonding systems. J Dent.

[17] Besnault C, Attal JP, Ruse D, Degrange M (2004). Self-etching adhesives improve the shear bond strength of a resin-modified glass-ionomer cement to dentin. J Adhes Dent.

[18] Khoroushi M, Mansouri T, Kamali B, Mazaheri H (2012). Marginal microleakage of resin-modified glass-ionomer and composite resin restorations: Effect of using etch-and-rinse and self-etch adhesives. Indian J Dent Res.

[19] Glasspoole EA, Erickson RL, Davidson CL (2002). Effect of surface treatments on the bond strength of glass ionomers to enamel. Dent Mater.

[20] Imbery TA, Namboodiri A, Duncan A, Amos R, Best AM, Moon PC (2013). Evaluating dentin surface treatments for resin-modified glass ionomer restorative materials. Oper Dent.

[21] Van Meerbeek B, De Munk J, Yoshida Y, Inoue S, Vargas M, Vijay P (2002). Buonocore memorial lectureAdhesion to enamel and dentin: current status and future challenges. Oper Dent.

[22] De Munck J, Van Landuyt K, Peumans M, Poitevin A, Lambrechts P, Braem M (2005). A critical review of the durability of adhesion to tooth tissue: methods and results. J Dent Res.

[23] Monticelli F, Toledano M, Silva AS, Osorio E, Osorio R (2008). Sealing effectiveness of etch-and-rinse vs self-etching adhesives after water aging: influence of acid etching and NaOCl dentin pretreatment. J Adhes Dent.

[24] El-Askary F, Nassif M (2011). Bonding nano-filled resin-modified glass ionomer to dentin using different self-etch adhesives. Oper Dent.

[25] Shafiei F, Akbarian S (2013). Microleakage of nanofilled resin-modified glass-ionomer /silorane- or methacrylate-based composite sandwich class II restoration: effect of simultaneous bonding. Oper Dent.

[26] Mitra SB, Lee CY, Bui HT, Tantbirojn D, Rusin RP (2009). Long-term adhesion and mechanism of bonding of a paste-liquid resin-modified glass-ionomer. Dent Mater.

[27] Coutinho E, Van Landuyt K, De Munck J, Poitevin A, Yoshida Y, Inoue S (2006). Development of a self-etch adhesive for resin-modified glass ionomers. J Dent Res.

[28] Frankenberger R, Lohbauer U, Roggendorf MJ, Naumann M, Taschner M (2008). Selective enamel etching reconsidered: better than etch-and-rinse and self-etch?. J Adhes Dent.

[29] Van Meerbeek B, Yoshihara K, Yoshida Y, Mine A, De Munck J, Van Landuyt Kl (2011). State of the art of self-etch adhesives. Dent Mater.

[30] Van Landuyt KL, Kanumilli P, De Munck J, Peumans M, Lambrechts P, Van Meerbeek B (2006). Bond strength of a mild self-etch adhesive with and without prior acid-etching. J Dent.

[31] Taschner M, Nato F, Mazzoni A, Frankenberger R, Kramer N, Di Lenarda R (2010). Role of preliminary etching for one-step self-etch adhesives. Eur J Oral Sci.

[32] Taschner M, Nato F, Mazzoni A, Frankenberger R, Falconi M, Petschelt A (2012). Influence of preliminary etching on the stability of bonds created by one-step self-etch bonding systems. Eur J Oral Sci.

[33] Fron H, Vergnes JN, Moussally C, Cazier S, Simon AL, Chieze JB (2011). Effectiveness of a new one-step self-etch adhesive in the restoration of non-carious cervical lesions: 2-year results of a randomized controlled practice-based study. Dent Mater.

[34] Bayrak S, Sen TE, Tuloglu N (2012). The effects of surface pretreatment on the microleakage of resin-modified glass-ionomer cement restorations. J Clin Pediatr Dent.

[35] Wang L, Sakai VT, Kawai ES, Buzalaf  MAR, Atta MT (2006). Effect of adhesive systems associated with resin-modified glass ionomer cements. J Oral Rehabil.

[36] Marquezan M, Fagundes TC, Toledano M, Navarro MF, Osorio R (2009). Differential bonds degradation of two resin-modified glass-ionomer cements in primary and permanent teeth. J Dent.

